# Upregulation of Nicotinic Acetylcholine Receptor alph4+beta2 through a Ligand-Independent PI3Kbeta Mechanism That Is Enhanced by TNFalpha and the Jak2/p38Mapk Pathways

**DOI:** 10.1371/journal.pone.0143319

**Published:** 2015-11-30

**Authors:** Scott W. Rogers, Lorise C. Gahring

**Affiliations:** 1 Salt Lake City Veteran’s Administration Geriatric Research, Education and Clinical Center, Salt Lake City, Utah, 84148, United States of America; 2 Department of Neurobiology and Anatomy, University of Utah School of Medicine, Salt Lake City, Utah, 84132, United States of America; 3 Department of Internal Medicine, Division of Geriatrics, University of Utah School of Medicine, Salt Lake City, Utah, 84132, United States of America; Universidade do Estado do Rio de Janeiro, BRAZIL

## Abstract

High affinity nicotine-binding sites in the mammalian brain are neuronal nicotinic acetylcholine receptors (nAChR) assembled from at least alpha4 and beta2 subunits into pentameric ion channels. When exposed to ligands such as nicotine, these receptors respond by undergoing upregulation, a correlate of nicotine addiction. Upregulation can be measured using HEK293 (293) cells that stably express alpha4 and beta2 subunits using quantification of [^3^H]epibatidine ([^3^H]Eb) binding to measure mature receptors. Treatment of these cells with choline also produces upregulation through a hemicholinium3 (HC3)-sensitive (choline kinase) and an HC3-insensitive pathway which are both independent of the mechanism used by nicotine for upregulation. In both cases, upregulation is significantly enhanced by the pro-inflammatory cytokine tumor necrosis factor alpha (TNFα) which signals through its receptor Tnfr1 to activate p38Mapk. Here we report that the inhibition of class1 phosphoinositide 3-kinases isoform PI3Kbeta using the selective antagonist PI828 is alone sufficient to produce upregulation and enhance both nicotine and choline HC3-sensitive mediated upregulation. Further, these processes are impacted upon by an AG-490 sensitive Jak2-associated pathway. Both PI3Kbeta (negative) and Jak2 (positive) modulation of upregulation converge through p38Mapk and both overlap with TNFalpha enhancement of this process. Upregulation through the PI3Kbeta pathway did not require Akt. Collectively these findings support upregulation of endogenous alpha4beta2 as a balance among cellular signaling networks that are highly responsive to multiple environmental, inflammatory and metabolic agents. The findings also suggest how illness and metabolic stress could alter the expression of this important nicotinic receptor and novel avenues to intercede in modifying its expression.

## Introduction

The addictive process to nicotine is in part modulated by the density and affinity of pentameric inotropic nicotinic acetylcholine receptors composed of alpha4 (α4) and beta2 (β2) subunits (α4β2; [1–3]). One mechanism contributing to the regulation of α4β2 receptor density after exposure to ligands such as nicotine is termed upregulation [3–8]. The cell biology and mechanisms underpinning upregulation are diverse and may involve components of subunit transcription and translation, receptor assembly, transport chaperones, surface expression of this receptor and changes in conformational state that promote high-affinity binding [3]. Upregulation is also promoted through other mechanisms that include exposure to receptor antagonists or indirectly through activation of cellular signaling networks that are independent of known α4β2-ligand interaction [1–3,8–10]. Our studies focus on understanding the cellular signaling pathways that modulate the upregulation through mechanisms independent of nicotine. This includes other agents such as choline, which is obtained through the diet, and the pro-inflammatory cytokine tumor necrosis alpha (TNFα), which significantly enhances nicotine or choline initiated upregulation processes.

Much of our understanding of upregulation has been derived from experimental examination using heterologous cell systems that express nicotinic receptors. One particularly successful experimental model that accurately reflects neuronal mechanisms employs HEK293 (293) cells that are stably transfected with the α4β2 receptors [6,7,9–13]. For example, similar to its effects on neurons, nicotine and other receptor ligands are potent inducer of upregulation in these 293 cells, which is measured by increased binding of the receptor-specific high affinity frog toxin, (^3^H)-epibatidine ([^3^H]Eb) to mature receptors in cell membrane preparations. In addition to nicotine, choline produces reliable upregulation through both a choline-kinase independent and dependent pathway that is distinguished by its sensitivity to inhibition by choline-kinase inhibitor, hemicholinium-3 (HC3). In this model system, upregulation is dominated through post-transcriptional mechanisms that increase β2 protein expression while maintaining a constitutively high level of α4 expression. Thus, as the β2 ratio is optimized to favor pentameric α4+β2 assembly, upregulation is achieved and enhanced further by the additional increase in β2 production promoted by TNFα [9,10]. In addition, enhanced upregulation by the pro-inflammatory cytokine TNFα imparts this effect through the tumor necrosis factor receptor 1 (Tnfr1) and signaling through the p38Mapk-dependent pathway which is sensitive to selective inhibition by SB202190 [9,14]. The upregulation produced by either choline and nicotine or its enhancement by TNFα is dramatically reduced when the alpha5 (α5) structural subunit is co-expressed with α4 and β2 (although α4β2α5 receptor densities are similar to upregulated α4β2 receptor [14] and see [15]). This result indicates that the influences on the upregulation process is a cell specific response that depends upon the combination of nAChR subunits expressed and the responsiveness to both agents such as dietary choline or the inflammatory status as reflected by TNFα and responsiveness to its signaling through Tnfr1.

In this study we have continued to examine the cell-signaling mechanisms that lead to ligand-independent α4β2 upregulation and/or its enhancement in the stably transfected 293 α4β2 cell culture model. This includes measurements of ligand-independent [^3^H]Eb binding upregulation in cells that were treated with cell-permeable small molecule inhibitors of the class1 PI3K activities [10]. Class 1 PI3Ks (e.g., [16,17]) are implicated in upregulation based upon the discovery that inhibition of their activity by the pan-inhibitors such as LY294002 directly produce upregulation and enhance both ligand (nicotine)-activated and choline-mediated upregulation [9,10,14]. The PI3K-upregulation requires inhibition of PI3Kβ, but not other isoforms, that acts through disinhibition of its effect on p38Mapk. In a distinct mechanism, enhancement of choline-mediated upregulation via the pathway, also leads to p38Mapk activation through upstream modification of the Jak2 activation and the positive modulation of p38Mapk. Thus endogenous upregulation pathways appear to converge through p38Mapk where upregulation is determined by the constitutive balance between choline-kinase HC3-dependent Jak2-mediated positive modulation and inhibition by PI3Kβ.

Collectively these results suggest that normal physiological maintenance of the α4β2 density reflects a balance between constitutive signaling through PI3Kβ, Jak2 and p38Mapk pathways that are subject to modification during dietary and pro-inflammatory events or possibly mostly bypassed by potent pro-upregulation agents such as nicotine. At present we find no evidence of an impact through these pathways on known proteins associated with α4β2 assembly and expression such as 14-3-3 or BiP, but through the use of a proteomic 2D-gel analysis preliminary evidence is presented to suggest additional proteins not presently associated with the process of upregulation may be contributing to the intracellular signaling networks controlling α4β2 protein subunit expression. The combined basal activity through these antagonistic mechanisms could control endogenous α4β2 expression and upregulation to influence the individual cell and physiological response to nicotine.

## Materials and Methods

### Cell lines and culture conditions

The HEK293 (293) cell line stably co-transfected with rat nAChR subunits α4 and β2 are well characterized [6,11,12], and they were maintained as described previously [9,10,14]. This cell line also expresses TNFR1 (only weak expression of TNFR2 is detected) and no additional human nAChR subunits or acetylcholine synthetic enzymes are detected [9,10]. As before treatments were conducted 48 hours after cell plating [9,10,14]. The source and optimal concentrations of the inhibitors used are as follows: From Sigma; 1 μM nicotine (nicotine hydrogen tartrate); 250 μM choline (choline chloride); hemicholinium-3 (HC3; 50 μM). From Biosource; 25 ng/ml human TNFα. From Tocris Bioscience: 1,2,3,4,5,6-Hexabromocyclohexane (1-6Hex; 50 μM), tyrphostin 2-cyano-3-(3,4-dihydrophenyl)-*N*-(phenylmethyl)-2-propenamide (AG-490; dose as in text), Cucurbitacin I (100 nM); GSK690693 (5 μM), Lestaurtinib (300 nM), Ly294002 (10 μM), PI 828 (20 nM), SB2020190 (10 μM), Wortmannin (3 μM), ZM 39923 (50 μM), ZM449829 (50 μM). From Cayman Chemical: AS-605240 (20–100 nM); PIK-75 (50 μM). Most inhibitors were dissolved in either DMSO or ethanol as the carrier and in all cases the carrier was added to cultures at the same concentration as before [9,10,14].

### Radioligand binding

Binding of [^3^H]-epibatidine ([^3^H]Eb; Perkin-Elmer Life Sciences) to crude cell membranes was as described previously [9–11]. Basically, cells were plated into 100 mm culture dishes for at least 48 hours before treatment. To harvest membranes, cells (18–24 hrs after treatment initiation) were rinsed in TBS, scraped into 50 mM Tris (pH 7.4), and disrupted in a glass Dounce homogenizer. Debris and nuclei were separated by low-speed centrifugation (300 xg) and the supernatant collected and pelleted by centrifugation at 20,000xg. The pellet was dissolved in a final concentration of 1% of TritonX-100, cleared by centrifugation and 5 μg of this soluble membrane fraction was incubated with 5 nM [^3^H]Eb for 2–4 h at room temperature. Binding assays were done in triplicate, and non-specific binding was measured in parallel tubes to which 300 μM nicotine hydrogen tartrate (Sigma) was added for 30 min prior to [^3^H]Eb. Samples were collected by vacuum filtrated through Whatman GF/C filters and then quantitated using standard scintillation counting. The specific binding was calculated by averaging the total binding minus the nonspecific (nicotine blocked) binding and analyzed using Prism 4 (v4.03, GraphPad Software Inc., San Diego).

### Western blot and cytochemical analysis

Antibodies to nAChR subunits [4,9,10,14] were rabbit polyclonal anti-α4 (5009 or 4964), anti-β2 (serum 4842 or 305). Other antibodies included immunological heavy chain-binding protein BiP (AbCam, ab21685), anti-14-3-3eta (AbCam, ab138463 or ab69591)), anti-annexin 2 (Novis, NBP1-31310), anti-prefoldin5 (Sigma-Aldrich, HPA008587) and anti-Ran (AbCam, ab31120). Western blots were prepared as described previously [9,10,14]. Briefly, treated cells were harvested, crude membranes prepared and dissolved in gel loading buffer containing dithiothreitol and heated to 95°C for 10–15 min. The samples were then fractionated by SDS-PAGE followed by semi-dry transfer to polyvinylidene difluoride membrane, blocked with 5% dry milk and 0.05% Tween 20 (PBS-T), and rocked overnight at 4°C in primary antibody. Blots were washed in Tris-saline, incubated with peroxidase-conjugated secondary antibody for 1 hour in blocking solution (at room temperature), washed, and then developed with the enhanced chemiluminescence system (PerkinElmer Life Sciences) immediately before preparing exposures on film. Multiple exposures were collected to assure linearity. Gels were recorded on a computer scanner, and the images were assembled using either Photoshop version 5.5 or Image Pro-plus (version 4.5, Media Cybernetics) software. Immunocytochemistry was performed with 2 percent paraformaldehyde fixation as before [18].

### 2D gel analysis

All methods, gel analysis and protein spot identification by Mass spectroscopy were performed according to direction provided by Applied Biomics Inc. according to their instructions. Briefly this was done by harvesting gently washing treated cells with 10 mM Tris-HCl, 5 mM magnesium acetate, (pH 8.0) and then scraping the cells from two 100 mm culture dish into 2 mls of this buffer and collected into Eppendorf tubes, and cells are pelleted by a 1-minute spin at low speed (100 xg). The supernatant was removed and frozen for shipment to Applied Biomics for analysis.

## Results

### Inhibition of PI3K produces upregulation of [^3^H]Eb sites on 293 cells stably transfected with nicotinic receptor α4 and β2 subunits

**Fig 1:**
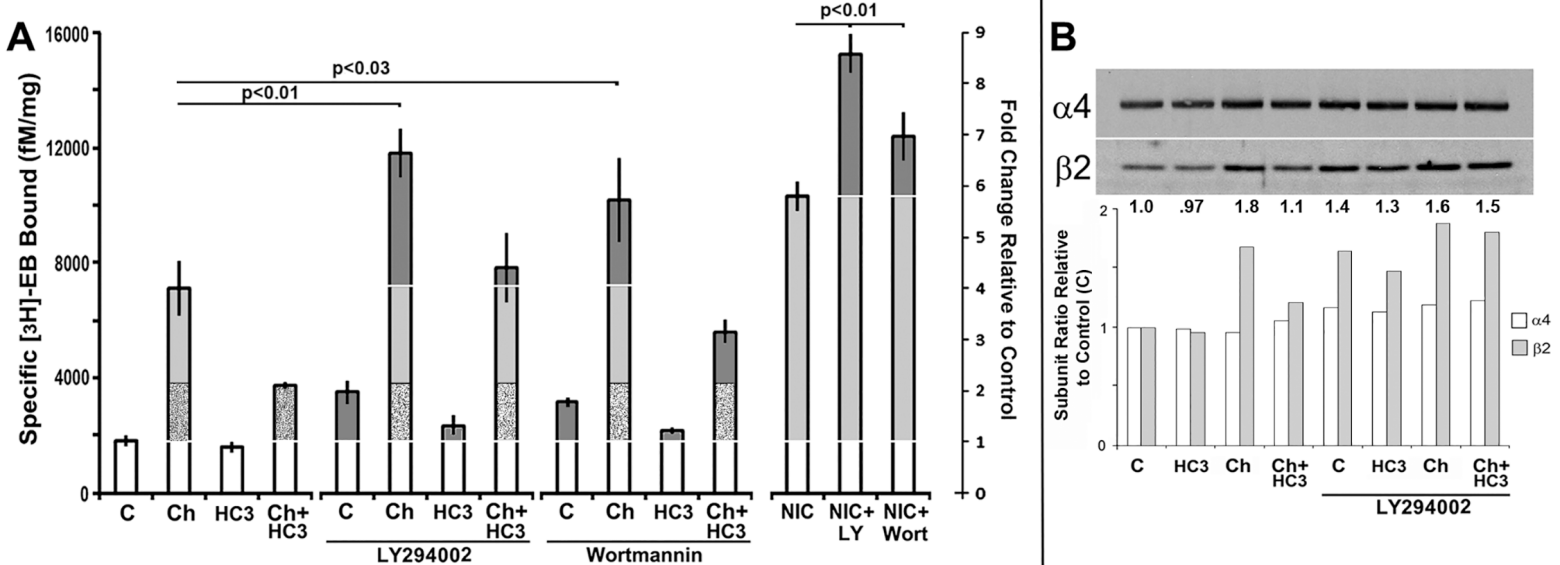
Inhibition of PI3K is sufficient to upregulate [^3^H]Eb binding and enhance choline HC3-sensitive upregulation. Stably transfected 293 cells were either not treated (C) or treated with choline (Ch), hemicholinium-3 (HC3) or both (Ch+HC3) and separately nicotine (Nic) either in the presence or absence of LY294002 (LY) or Wortmannin (Wort), respectively. **A)** Cells were harvested 24 hours post treatment and specific binding by the α4β2 high affinity ligand [^3^H]Eb was measured (Methods). In all cases the results reflect the average of no less than 3 independent experiments. Upregulation is indicated in the light grey, enhancement of upregulation is in dark grey and stippled is the HC3-insensitive choline component. Error bars = +/- SEM. The significance values (P) are calculated from Student’s t-test of the indicated pairing. **B)** Western blot analysis of crude membranes prepared from choline-treated cells that received treatment as labeled and corresponded to those used for [^3^H]Eb binding to measure the relative expression of α4 or β2 subunits. The ratio of the band density for each subunit was measured, normalized to the control (1.0) and then plotted for each treatment. Above each lane is the change in the β2/α4 ratio calculated after normalization. The blot shown reflects a result that is typical for these experiments.

In previous reports we noted that the potent class I phosphoinositide 3-kinases (PI3K) pan-inhibitor LY294002 was alone sufficient to produce significant upregulation of the ^3^H-epibadidine ([^3^H]Eb) sites associated with increased receptor expression in 293 cells stably transfected with the nicotinic acetylcholine receptor subunits α4 and β2 [9,10,14]. Also, in response to elevated choline or nicotine, the co-application of LY294002 greatly enhanced the upregulation of [^3^H]Eb produced by either agent alone. This result was confirmed and extended by measuring the effect of two widely used and well characterized as pan-inhibitors of class1 p110 PI3K catalytic activity; LY294002 and Wortmannin [16,19,20], on upregulation both alone and in the presence of choline (Fig 1A). Both inhibitors promoted upregulation of [^3^H]Eb binding in the absence of receptor ligand. Because the LY294002 effect was more consistent with less impact on cell morphology compared to wortmannin (Fig 1A and not shown), this agent was used for essentially all of the subsequent experiments. Choline alone produces upregulation through a hemicholinium-3 (HC3) sensitive, choline kinase (CK)-dependent [21], and a HC3-insensensitive pathway [10], we examined which of these pathways was most impacted by PI3K. The results reveal that LY294002-associated upregulation enhances choline-mediated upregulation of [^3^H]Eb. Further, both the HC3-sensitive and insensitive choline mediated upregulation pathways are enhanced by LY294002 and to a lesser extent by Wortmannin. In all cases, as before inhibition of PI3K activity using LY294002 or Wortmannin also enhances nicotine mediated upregulation (Fig 1A). These results are consistent with the conclusion that constitutive PI3K activity is a constitutive inhibitor of upregulation.

One way to produce upregulation is to produce optimal assembly ratios of each subunit through increasing β2 subunit expression in the presence of excess α4 to enhance the production of mature receptors and increased [^3^H]Eb binding [9,10]. To determine if inhibition of PI3K promotes upregulation though increased β2 translation, western blot analysis was used (Fig 1B). Cells that were treated for 24 hours with the PI3K inhibitors, harvested and membranes prepared for subunit expression analysis using western blot for α4 or β2 expression, respectively (Methods and [9,10,14]). The result show that upregulation of [^3^H]Eb binding following inhibition of PI3K corresponded with an increase in the expression of β2 with little impact on the constitutive and relatively high production of α4. As before [9] inhibition of protein synthesis in this system inhibited the increase in [^3^H]Eb upregulation (not shown). Quantitation of these blots for the relative α4 or β2 expression (Fig 1B) produced results that were also consistent with prior studies [9,10,14] where optimal [^3^H]Eb binding corresponded to an increased amount of β2 and a relatively constant level of α4 expression. Of note is that the LY294002-associated increase in β2 subunit expression persists despite inhibition of the HC3-sensitive component of choline mediated upregulation (compare Fig 1A to 1C). One explanation for these results is that PI3K inhibition on [^3^H]Eb upregulation begins in parallel with choline-dependent upregulation mechanisms and intercepts at a downstream site whereas enhancement of the impact of nicotine is largely independent and converges at the point of receptor assembly.

### Inhibition of the PI3Kβ isoform produces ligand-independent [^3^H]Eb receptor upregulation and enhances upregulation by both choline and nicotine

**Fig 2:**
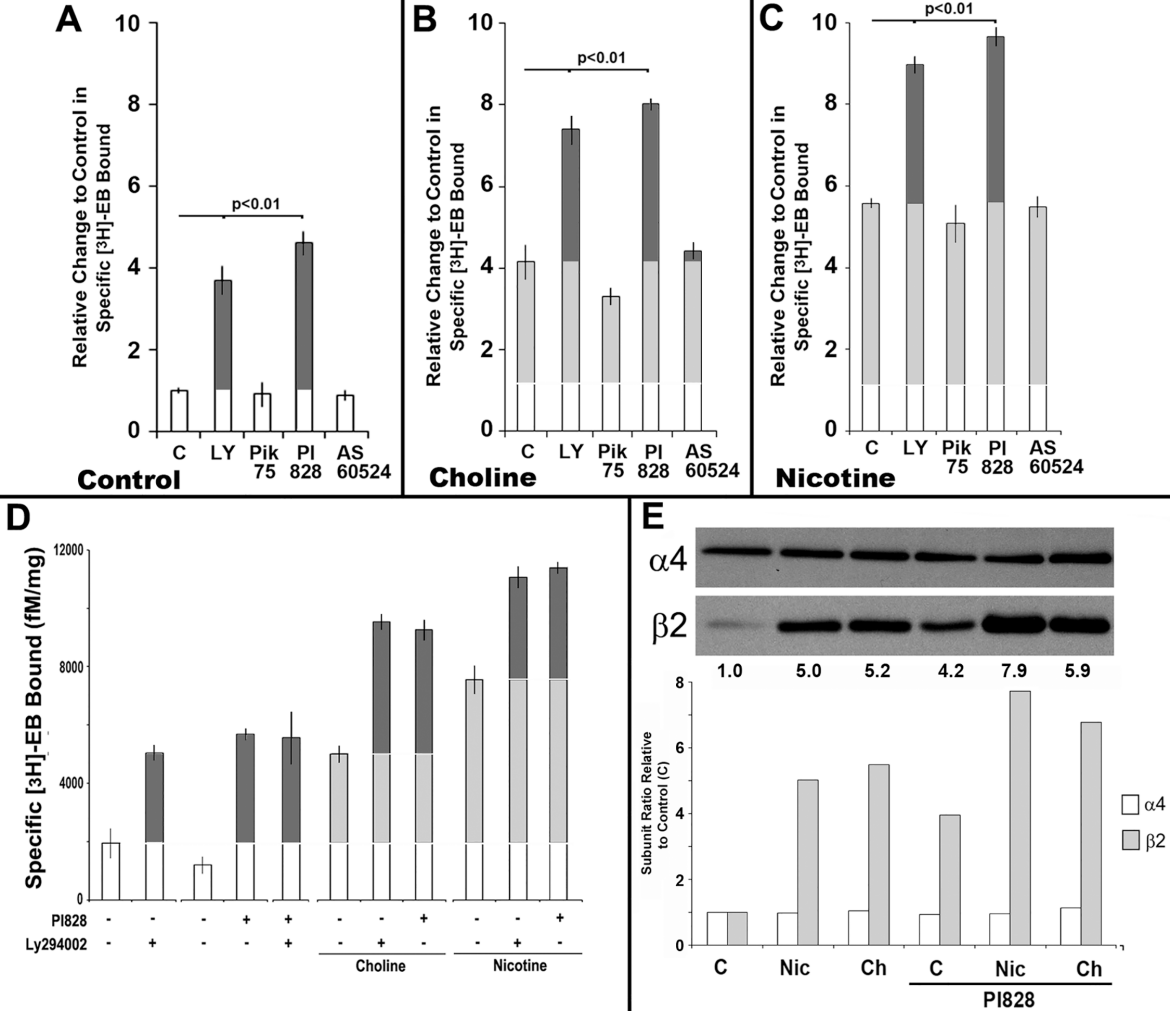
Inhibition of the PI3Kβ isoform produces ligand-independent [^3^H]Eb receptor upregulation and enhances upregulation produced by choline and nicotine. **A-C)** The results from measurements of stably transfected 293 cells that were either not treated (C) or treated with the PI3K inhibitors LY294002 (LY, broad range), PIK75 (PI3Kα), PI828 (PI3Kβ), or AS60524 (PI3Kγ) as in **(A)**, or in the presence of **(B)** choline or **(C)** nicotine as normalized to the control in (A). The specific [^3^H]Eb binding to membranes from these cells was measured for 3 independent experiments, the results normalized to the average no-treatment control value of 1.0 and all values from each experiment were then summed. **D)** Experiments similar to those in Panels A-C tested the impact to combining LY294002 and PI828 on upregulation of [^3^H]Eb binding or enhancement of upregulation produced by either choline or nicotine as indicated by + signs indicating addition of the agent. Upregulation relative to the control for each group is in light grey and enhancement of choline or nicotine upregulation is in dark grey. Error bars = +/- SEM. The significance values (P) are calculated from Student’s t-test of the indicated pairing. **E)** An example of a representative western blot showing the relative expression of α4 or β2 subunits in crude membrane fractions of cells after receiving the treatment as labeled. Nic, nicotine; Ch, choline. The ratio of the band density for each subunit was measured, normalized to the control (1.0) and then plotted for each treatment. Above each lane is the change in the β2/α4 ratio calculated after normalization.

Four major PI3K isotypes [16,17] regulate many cellular processes including cell growth and survival as well as vesicular and membrane trafficking including p110α and p110β (both widely expressed) or the more restricted expression of p110γ and p110δ (which is largely hematopoietic). To define which class1 PI3K isoform(s) promote upregulation, isoform-selective inhibitors were tested for their impact on PI3K-mediated upregulation of [^3^H]Eb binding (Fig 2). The 293 cells stably expressing α4 and β2 subunits were treated with different PI3K-specific inhibitors that included PIK-75 for PI3Kα [22], PI828 for PI3Kβ [23], and AS605240 for PI3Kγ [24]. Due to hematopoietic-preferred expression, PI3Kδ was not examined in these 293 cells (not shown). The results show that inhibition of PI3Kβ by PI828 was alone sufficient to impart upregulation whereas inhibitors of PI3Kα and PI3Kγ had no effect (Fig 2A). In some experiments the inhibition of PI3Kα by PIK75 could be associated with decreased α4β2 binding sites; although this effect was dose-dependent and increased as inhibitor concentrations reached 10x over the optimal KI (not shown). The use of additional inhibitors more specific to PI3Kα (e.g., PI-103; [22]) did not alter control level [^3^H]Eb binding (not shown). Thus, regulation of the α4β2 receptor expression and binding upregulation in the absence of ligand requires the PI3Kβ isoform.

Next it was determined if the inhibition of PI3Kβ alone or in combination with other PI3K isoforms modifies either choline or nicotine mediated upregulation. In these experiments parallel sets of stably transfected 293 cells were established and receiving a PI3K inhibitor as above, but some cultures also received co-treated with either choline and PI3K inhibitor, or nicotine and PI3K inhibitor. The average results from at least 5 experiments for each condition are shown in Fig 2B and 2C. Upregulation was enhanced by either the PI3K broad range LY294002 inhibitor or an equivalent amount by PI828 inhibition of PI3Kβ alone. The inhibition of other isoforms failed to impact on upregulation produced by either just choline or nicotine, respectively. To determine if LY294002 and PI828 interact, these agents were added separately or together and in the presence and absence of either choline or nicotine (Fig 2D). The co-application of LY294002 and PI828 produced effects equivalent to either agent alone. Thus, collectively the results strongly support a model where the specific inhibition of PI3Kβ is alone sufficient to promote upregulation of [^3^H]Eb binding or enhance upregulation produced by either choline or nicotine.

To determine if choline or nicotine enhanced [^3^H]Eb binding upregulation corresponded to increased β2 subunit expression, we repeated the experiments performed above only this time cells were prepared for western blot analysis. As shown in Fig 2E, in all cases PI828 treatment was alone sufficient to increase β2 expression (bar graph for the experiment shown) and this coincided with increased [^3^H]Eb binding (Fig 2D). Further, the increase in β2 produced by choline or nicotine alone was enhanced even further by PI828. In all cases, as reported previously [9,10,14] there is essentially no change in the expression of α4 that is related to either choline or nicotine. When the ratio of α4:β2 was calculated from quantification of western blot results (Fig 2E; [10]), [^3^H]Eb binding was consistent with the increase and apparent excess of β2 subunit relative to α4. This is again consistent with the findings that optimization of the α4:β2 ratio, especially as β2 is produced in excess of α4, favors increased [^3^H]Eb binding sites (not shown; see [10]). Collectively, these experiments favor the model in which constitutive PI3Kβ activity normally leads to the suppression of [^3^H]Eb upregulation through suppression of β2 subunit translation, stability and/or other routes of accomplishing maturation into mature receptors.

### The HC3-sensitive, but not the HC3-insensitive pathway to choline–mediated upregulation requires p38Mapk

**Fig 3:**
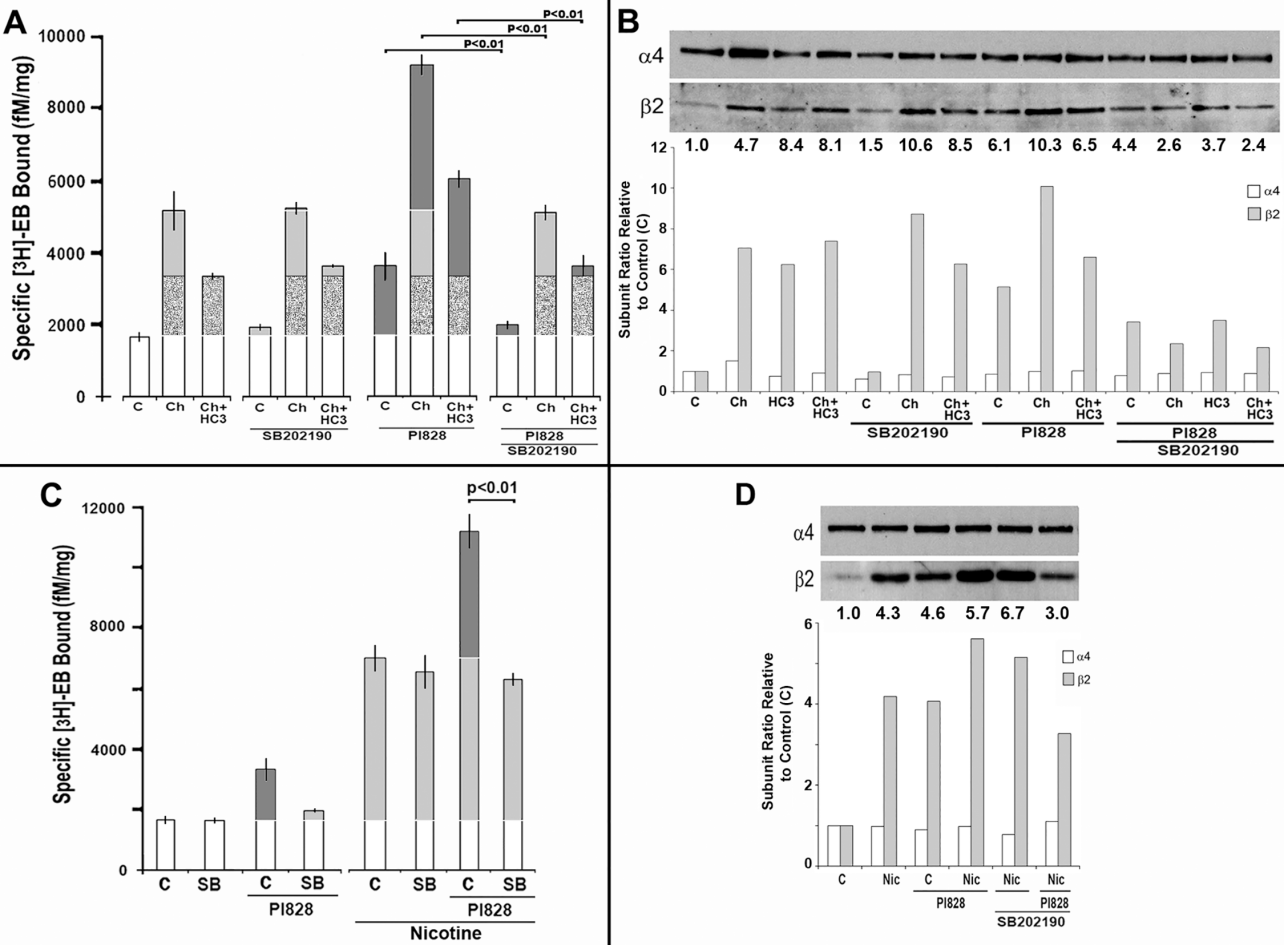
Upregulation related to inhibition of PI3Kb requires p38Mapk. **A)** Stably transfected 293 cells were either not treated (C) or treated with choline (Ch), or choline + hemicholinium-3 (HC3) in the presence of the p38Mapk inhibitor SB202190, the PI3Kβ inhibitor PI828 or both inhibitors as indicated. Specific [^3^H]Eb binding to crude cell membranes was measured at 24 hours post-treatment. Upregulation due to choline or nicotine is indicated in light grey and the ‘enhanced’ component in dark grey. Each bar is the average of no less than 3 independent experiments and error bars = +/- SEM. The significance values (P) are calculated from Student’s t-test of the indicated pairing. HC3 alone, as in other experiments, had not significant effect (data not shown). **B)** Western blot analysis of crude membranes prepared from cells in parallel with experiments in panel A. The ratio of the band density for each subunit was measured, normalized to the control (1.0) and then plotted for each treatment. Above each lane is the β2/α4 ratio after normalization. The blot shown reflects a typical result for these experiments. **C)** The results of experiments performed in parallel with those in panel A and B only nicotine was substituted before measuring (C) [^3^H]Eb binding or (D) western blots of α4 or β2.

Choline-mediated upregulation is the summation of two processes distinguished by sensitivity to inhibition of choline kinase (CK) catalytic activity by hemicholinium-3 (HC3; [9]). Because CK impacts both p38Mapk and PI3K/Akt signaling [25], the possibility of interactions between PI3Kβ and the HC3-sensitive or HC3-insensitive [^3^H]Eb upregulation pathways, including acting through p38Mapk, were investigated. Cultures were prepared in parallel that received no treatment, choline, HC3 or choline plus HC3. As shown in Fig 3A and 3B and as reported previously [9,10], approximately 50–60% of the choline-mediated upregulation was sensitive to inhibition by HC3. Treatment with HC3 alone or in combination with other inhibitors such as PI828 produced no differences relative to the respective controls (not shown). In this and the experiments to be described, modification to the expression of β2 and alteration of the α4:β2 ratio was observed and it was again consistent with mechanism leading to altered [^3^H]Eb binding (Fig 3B).

We then determined if inhibition of p38Mapk by SB2020190 or PI3Kβ by PI828, respectively, modified the choline HC3-sensitive, HC3-insensitive or both pathways leading to upregulation. Using the same approach as above, [^3^H]Eb binding was measured in transfected cells that were also co-treated with SB202190, PI828, or both agents together. As expected the PI828 inhibitor alone produced upregulation and it substantially enhanced choline-mediated upregulation. However, in the presence of HC3, the HC3-sensitive and enhanced upregulation contributed by PI828-inhibition of PI3Kβ was lost, resulting in [^3^H]Eb binding that was equivalent to the summation of the individual contributions by PI828 and the HC3-insensitive components. When SB202190 was co-applied with PI828, a notable effect was the loss of [^3^H]Eb binding upregulation produced by inhibition of PI3Kβ (Fig 3A). Further, inhibition of p38Mapk by SB202190 also decreased the PI828-mediated up-regulation component that remained in cells treated with PI828 + Choline + HC3, leaving only the [^3^H]Eb binding sites measured in the HC3-insensitive component of choline-mediated upregulation. Similar results were obtained when LY294002 was used in place of PI828 (not shown and below). Thus, the HC3-insensitive component of choline upregulation does not require p38Mapk and it does not overlap with the pathways influenced by PI3Kβ inhibition. However, both upregulation of [^3^H]Eb binding produced by inhibition of PI3Kβ alone or enhancement of choline mediated HC3-sensitive upregulation converge through a common p38Mapk pathway.

The sensitivity of PI3Kβ inhibition by PI828 and the corresponding increase in nicotine-mediated upregulation through disinhibition of p38Mapk was also examined (Fig 3C and 3D). Similar to the results for choline in Fig 3A, inhibition of PI3Kβ by PI828 produced upregulation and enhancement of the robust upregulation produced by nicotine. However, in all cases co-application of SB202190 inhibited both PI828 upregulation and enhancement of nicotine upregulation without an impact upon controls or nicotine associated upregulation. Also, the increase in β2 produced by nicotine and enhanced by PI828 was abolished by SB20230 inhibition of p38Mapk (Fig 3D). This decrease was somewhat below that observed for nicotine alone although this did not reach statistical significance (not shown). Thus, these data indicate a common pathway leading to enhancement of upregulation through p38^Mapk^ is down-stream of PI3Kβ and that under constitutive conditions PI3Kβ appears to inhibit the p38^Mapk^ activity associated with altered β2 expression and [^3^H]Eb binding levels.

### TNFα enhancement and PI3Kβ inhibition of upregulation both act through p38Mapk

**Fig 4:**
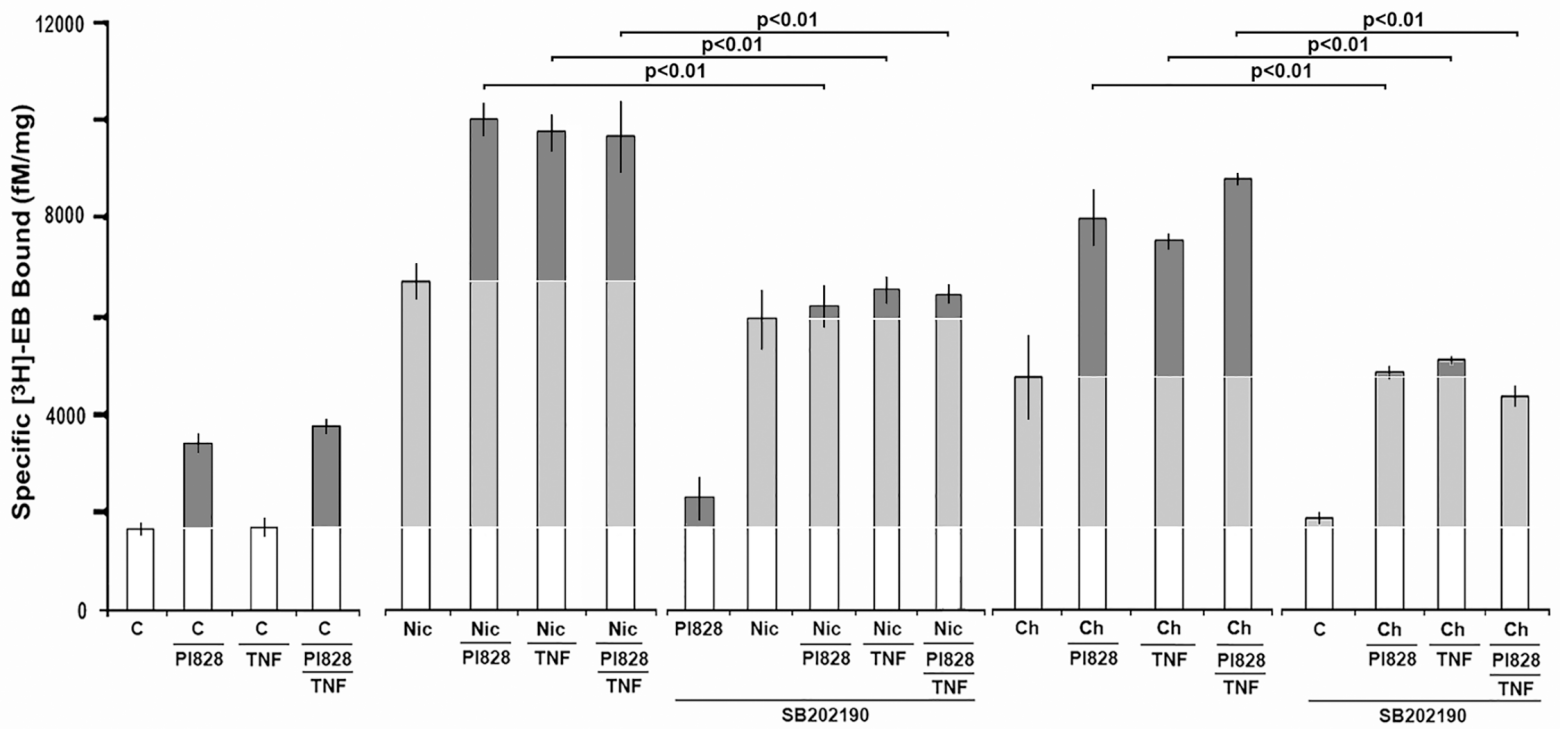
TNFα enhancement and PI3Kβ inhibition of upregulation both act through p38Mapk. Experiments similar to those in Fig 3 were performed with the addition of treatment of cells with nicotine (Nic) or choline (Ch) and each with PI828, TNFα or both as indicated. At 24 hours post-treatment specific [^3^H]Eb binding by crude cell membranes was measured. Each bar is the average of no less than 3 independent experiments and error bars = +/- SEM. The significance values (P) are calculated from Student’s t-test of the indicated pairing. Upregulation is indicated in the light grey and enhancement of upregulation is in dark grey.

In previous reports we demonstrated that TNFα through activation of TNFR1 leads to p38Mapk activity and the strong enhancement of α4β2 receptor ligand and choline-mediated upregulation [9,10,14]. This is similar to the results in Fig 3 where the enhancement of nicotine or the HC3-sensitive component of choline-mediated upregulation by inhibition of PI3Kβ is itself inhibited by the block of p38Mapk by SB202190. However, PI3Kβ inhibition differs in that it is alone sufficient to produce limited upregulation whereas TNFα does not do this [9,10,14]. Nevertheless the convergence of these activates at p38Mapk is notable and suggests that PI3Kβ activity as a negative upstream regulator of p38Mapk would also impact upon TNFα/TNFR1 singling in this system. To test this possibility, transfected 293 cells were treated as above with various combinations of PI828 with or without co-applications of TNFα, choline and nicotine, respectively. The results of these experiments, which in part also overlap with and reproduce those of Fig 3, are shown in Fig 4. As anticipated, PI828 alone produces an approximately 2-fold increase in upregulation but also in the presence of either nicotine or choline where upregulation is significantly enhanced over the effect of either of these agents alone. When TNFα is co-applied with nicotine, upregulation is increased from ~5.5-fold to ~8-fold or for choline from ~4-fold to ~6.5-fold. The same results were also obtained when nicotine or choline were combined with PI828 and/or TNFα, respectively (Fig 4). When the same experimental treatments included co-application of SB202190, all enhancements of nicotine or choline [^3^H]Eb binding produced by PI828, TNFα or a combination of these was lost (Fig 4). Thus, the conclusion is supported that both enhanced upregulation by either TNFα or PI3Kβ inhibition both require and act through p38Mapk.

### TNFα enhancement and HC3-sensitive choline [^3^H]Eb upregulation requires Janus kinase 2

The conclusion of a simple interaction of PI3Kβ and TNFα at p38Mapk is likely to be too simple. This is because PI828 inhibition of PI3Kβ alone produces upregulation whereas TNFα acting through TNFR1 leads only to enhancement of upregulation by choline or nicotine. Because our findings indicate the potential of overlap between PI3Kβ and TNFα, we examined the impact of inhibition of the Janus kinase (Jak)-dependent pathways on upregulation and enhancement produced by PI3Kβ inhibition. While the Jak pathways is often associated with α7 nAChR [26,27], other receptors including α4β2 also modulate these kinases [28]. Also, the impact of α4β2 on suppressing inflammation has been reported to proceed through a Jak2-depedent pathway [29]. The Jak pathway is also of interest because it is crucial to TNFα-mediated activation of p38Mapk through TNFR1 and it is likely to work upstream of the common mediator, p38Mapk [9]. Therefore, we examined the impact of Jak inhibitors on enhancement of upregulation by choline or nicotine upregulation by TNFα.

**Fig 5:**
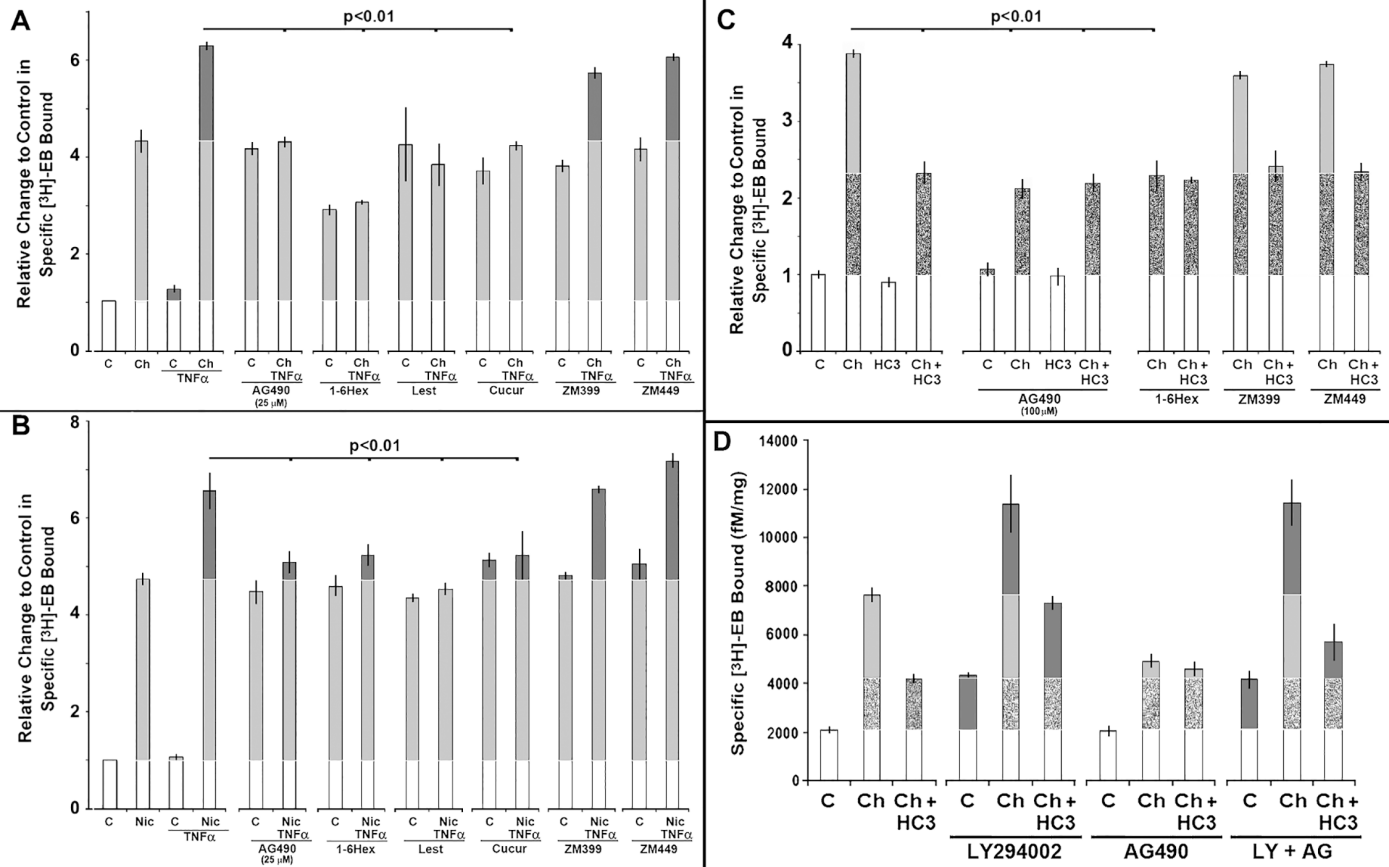
Upregulation of HC3-sensitive choline [^3^H]Eb binding and TNFα enhancement of this process requires Janus kinase 2. Stably transfected 293 cells were exposed to **(A)** choline, Ch, or **(B)** nicotine, Nic, with or without addition of TNFα (25 ng/ml). In parallel cultures, inhibitors with differing specificities towards members of the Jak family were also added. The agents used were 1,2,3,4,5,6-Hexabromocyclohexane (1-6Hex; 50 μM); AG-490 (AG490, 25 μM), cucurbitacin I (Cucur, 100 nM); lestaurtnib (Lest, 300 nM), ZM 39923 (ZM399, 50 μM), ZM449829 (ZM449, 50 μM). For all plots the error bars are +/- SEM and upregulation is indicated in the light grey and enhancement of upregulation is in by dark grey. The significance values (P) are calculated from Student’s t-test of the indicated pairing. **C)** Experiments similar to those in panels A examined the impact of Jak inhibitors on choline-mediated upregulation. C, control; Ch, choline; HC3, hemicholinium-3. For this experiment the concentration of AG-490 was 100 μM (see text), but all other inhibitors were used as above. For choline upregulation, solid light grey is HC3-sensitive and light grey is HC3-insensitive. **D)** The impact of co-application of inhibitors of PI3K and those favoring Jak2 are shown. Experiments were conducted as described in prior figure legends and the text. The stippled area is equivalent to the HC3-independent choline mediated upregulation and the light grey is the HC3-dependent component. Dark grey is the component of upregulation associated with the indicated treatment.

Multiple inhibitors with varied specificity towards Jak1, Jak2 or Jak3 were examined. A particularly widely used and well-characterized inhibitor of Jak2 is the tyrphostin AG-490 [30]. Although this inhibitor is often viewed as specific to Jak2, at concentrations that achieved optimal Jak2 inhibition there could be additional off-site effects on other tyrosine kinase activities (e.g., EGFR;[31]). Given this caveat, we started by examining the impact of AG-490 towards choline or nicotine upregulation or its enhancement by TNFα. Transfected 293 cells were treated with either choline with or without TNFα as a control. As shown in Fig 5A, the anticipated increase in upregulation was achieved by both of these agents and they are enhanced further by TNFα. In the presence of AG-490 (1 to 25 μM, 10 μM shown), all TNFα-mediated enhancement of choline or nicotine upregulation was abolished with no effect on the upregulation by either agent alone.

The specificity of AG-490 antagonism of TNFα was examined further using additional inhibitors of varied specificities towards the Jak family members (Fig 5). These included 1,2,3,4,5,6-hexabromocyclohexane (1-6Hex, 60 μM; Jak2-specific;[32]), lestaurtnib (300 nM; Jak2/Stat5-favoring; [33]), Cucurbitacin 1 (100 nM Jak2/Stat3 favoring; [34]), ZM39923 (50 μM; favoring Jak1/3;[35]), ZM449829 (50 μM; favoring Jak3;[35]). The results in Fig 5A and 5B show that the inhibitors specific to or favoring Jak2, but not those favoring Jak1 and/or Jak3, inhibited TNFα-mediated enhancement of both nicotine and choline upregulation. The possibility that these agents would also act through inhibition of EGFR at the concentrations does not seem likely since there is no impact by any of these potential on upregulation by choline, nicotine or PI828. This was confirmed in a separate experiment where the highly specific EGFR inhibitor AG-1478 (1 μM) produced no change in [^3^H]Eb binding (not shown). Collectively these results place Jak2 in the pathway through which TNFα promotes enhanced upregulation.

During the course of these experiments it was noted that the inhibitor, 1-6Hex (Fig 5C) produced partial inhibition of choline-mediated upregulation that was not produced by AG-490 at the concentrations used. A dose-response measurement of the impact of AG-490 on choline-mediated upregulation (not shown) revealed that at final concentrations of 100 μM to 300 μM, which are commonly reported in the literature for this tyrphostin, inhibited at least 50% of upregulation to choline (Fig 5C). The co-application of HC3 had no further impact indicating the HC3-sensitive component of choline-upregulation was affected (Fig 5C). This was also true of the other Jak2 inhibitor, 1-6Hex. As expected that inhibitors of Jak1/3 (ZM39923) or Jak3 (ZM449829) had no impact on choline-mediated upregulation (Fig 5C). Thus the results support the conclusion that Jak2 is also a component of the HC3-sensitive, choline kinase-dependent upregulation pathway.

The preceding results support a role for Jak2 in promoting choline HC3-sensitive upregulation, which is also the pathway through which PI3Kβ inhibits this same process. This suggested that simultaneous inhibition of PI3Kβ and Jak2 could be complementary and restore normal choline-mediated upregulation or, if PI3Kβ and Jak2 are in sequential pathways, either enhance or block their respective impacts on upregulation depending upon their order of impact on p38Mapk (Fig 4). To test this, parallel cultures either receiving no addition, choline or choline + HC3 were treated with AG-490, LY294002 or both, respectively. Membranes from the treated cells were then harvested and [^3^H]Eb binding measured. The results of these experiments are shown in Fig 5D. As expected from the results of previous experiments (Figs 1, 2 and 3), inhibition of PI3Kβ using LY294002 alone produced upregulation and enhanced this process in the presence of choline. Similarly, as predicted by the results in Fig 5C, inhibition of Jak2 using AG-490 inhibited the HC3-sensitive component of choline upregulation. When the agents were co-applied, the results were equivalent to LY294002 inhibition of PI3Kβ. This demonstrates that the inhibition of PI3Kβ by LY294002 was not blocked (or enhanced) by AG-490 suggesting that release of p38Mapk activity by inhibition of PI3Kβ is not additive with, or dependent upon, Jak2 activity. Thus, consistent with our findings the promotion of p38Mapk activation by Jak2 is tempered by convergence of the dominant inhibitory modulation imparted by PI3Kβ activity.

### Upregulation through disinhibition of PI3Kβ does not require phosphorylation of Akt

**Fig 6:**
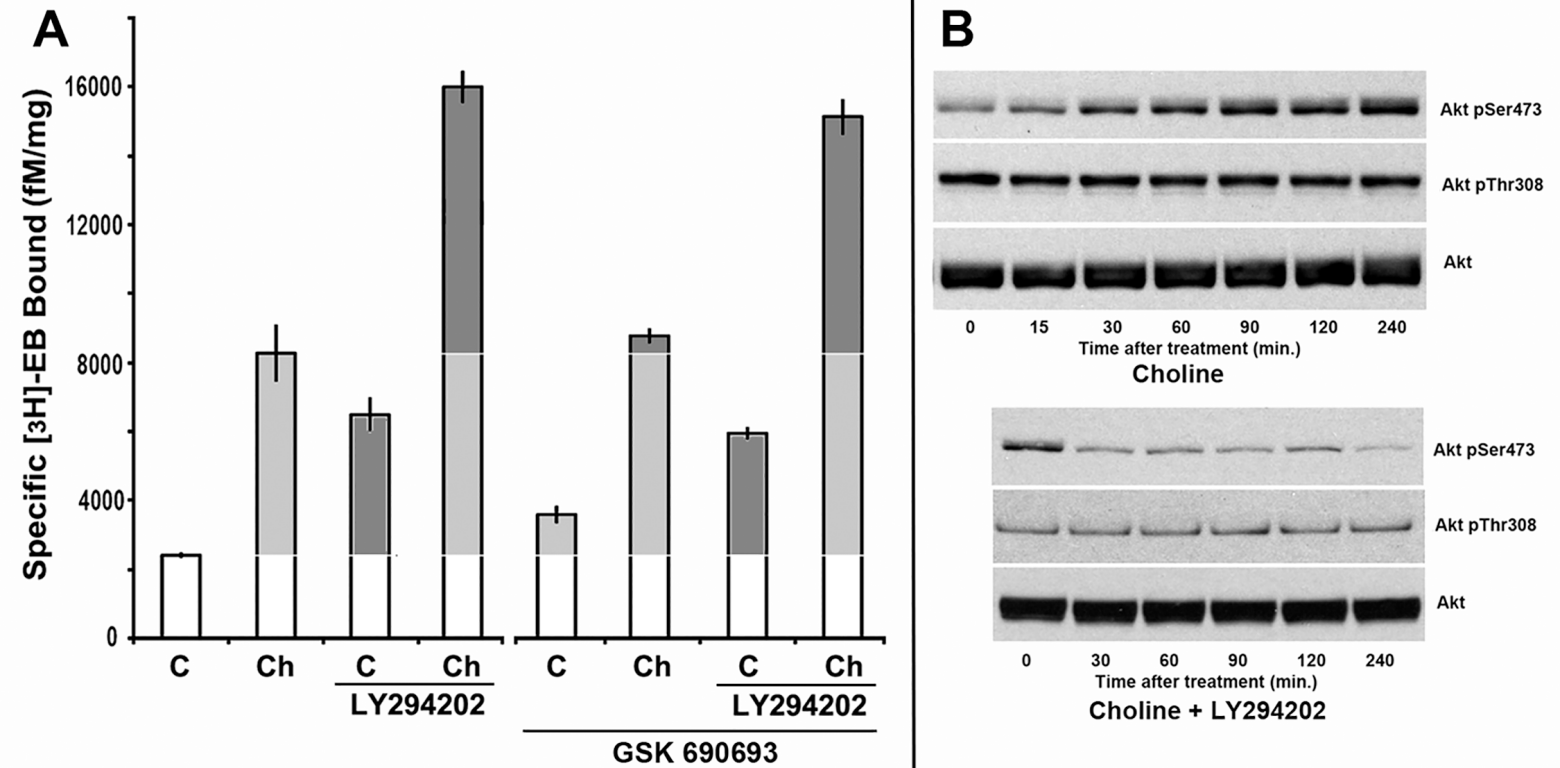
Upregulation through disinhibition of PI3Kβ does not require AKT. Cultures of stably transfected 293 cells were treated with choline (Ch) or controls that (C) received no treatment. To parallel culture sets either the PI3K inhibitor LY294202 or the Akt inhibitor GSK690693 was either added or co-applied with the other agents as indicated. Cultures were harvested and the specific [^3^H]Eb binding measured. The error bars = +/- SEM and upregulation is indicated in the light grey and enhancement of upregulation is marked as dark grey. Western blot analysis of 293 cells receiving the treatment indicated were harvested at the time indicated post-addition of the treatment agents and assayed for the expression of total Akt (Akt) or antibodies specific to Akt phosphorylated at either Ser473 (Akt pSer473) or Thr308 (Akt pThr473).

An important target of the products of PI3K signaling is Akt (protein kinase B) and PDK1 [36]. Although Akt is not necessarily a major target of the PI3Kβ isoform [37–39], there remains the possibility this important kinase activity serves as a down-stream target of PI3Kβ. Further, choline promotes activation of Akt in human breast carcinoma cells through CK-mediated enhancement of Akt phosphorylation at Ser473 (Akt^pSer473^;[40]). Thus, the suggestion that alterations in phosphorylation of Akt by inhibition of PI3Kβ correspond with or contribute directly to upregulation mechanisms was examined. Cultures of stably transfected 293 cells were established as described in previous experiments. One set of cultures received the Akt inhibitor, GSK690693 (1 μM; [41]) prior to the addition of either choline or LY294002. LY294002 was used to assure additional specificity of the PI3Kβ requirement since it is the PI3Kα isoform that dominates the Akt activation pathway whereas only a small if any contribution is made by PI3Kβ [38]. The results (Fig 6) reveal no evidence of an effect by Akt inhibition on upregulation. Also as part of the controls to measure Akt activity, western blot analysis of the phosphorylation of Akt^pSer473^ or another phosphorylation site, Akt^pThr308^, was used. Cell cultures were prepared in parallel, treated and then at various times thereafter (15, 30, 60, 90, 120, and 240 minutes, respectively) they were quickly rinsed in PBS and scraped into harvesting buffer (Methods). Cell lysate was then prepared for western blot analysis to measure the relative change in the total Akt, or phosphorylation of Akt at either Akt^pThr308^ or Akt^pSer473^, both markers of Akt activation. As shown in Fig 6B, the addition of choline produced an increase in Akt pSer473 phosphorylation that was evident within 15 minutes. The increase in phosphorylation continued steadily until reaching a maximum by 90 minutes as has been reported by others [41]. Choline addition did not result in modification of the phosphorylation status of Akt^pThr308^. In the presence of the pan-inhibitor of PI3K, LY294002 (10 μM), the choline mediated increase in Akt^pSer473^ was rapidly reduced and nearly absent 2 hours after initiating this treatment. A change in phosphorylation of Akt^pThr308^ was not observed. Despite the substantial change in upregulation produced by choline, LY294002 alone or both agents together (Fig 6A), the phosphorylation status of Akt at the sites measured did not correlate with nor reflect the robust changes in upregulation being measured. Thus, no evidence of an effect by Akt activation or inhibition on upregulation was identified.

### Choline-mediated upregulation does not correspond with a change in chaperone proteins 14-3-3eta or BiP, but may involve novel proteins identified by 2D-gel analysis

**Fig 7:**
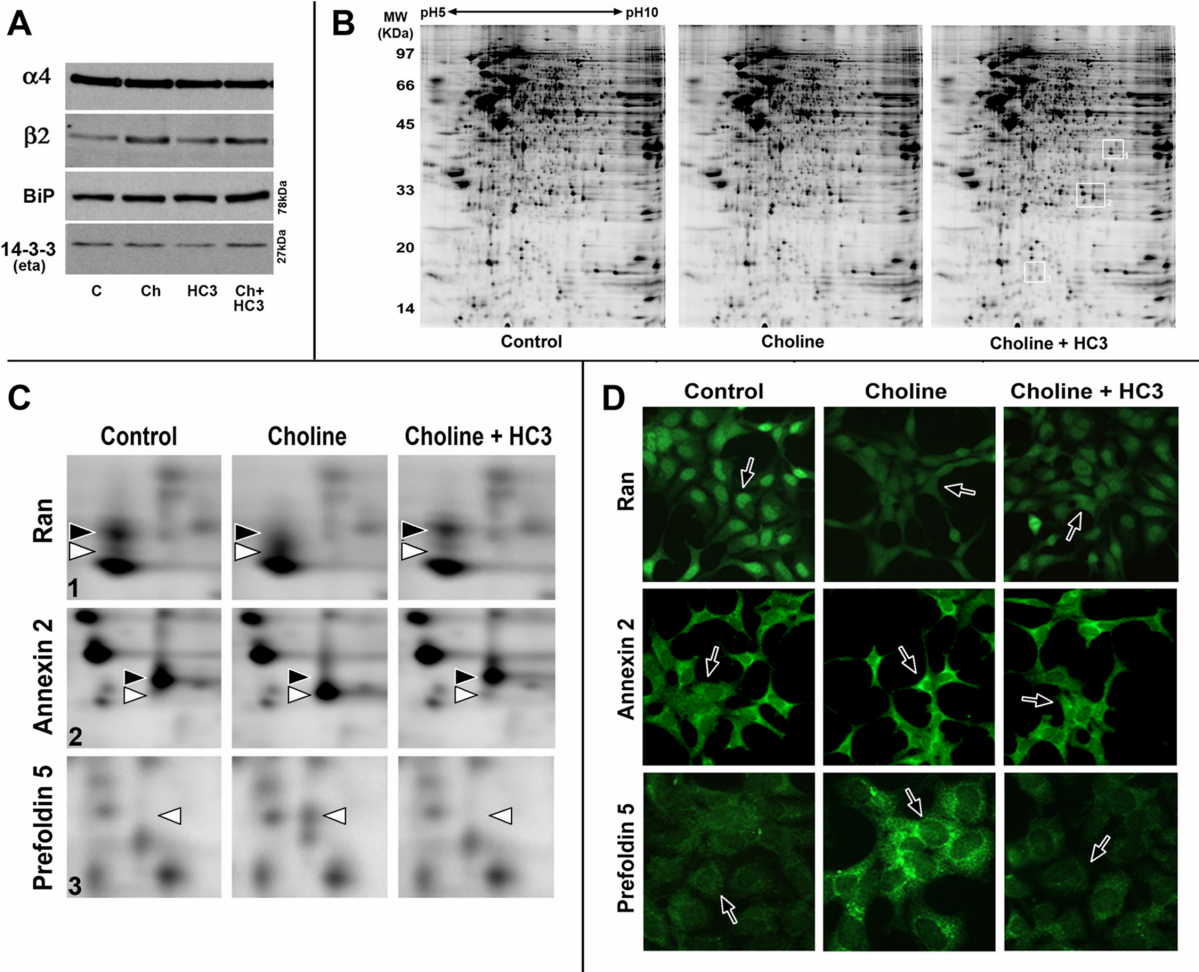
Choline-mediated upregulation does not correspond with a change in chaperone proteins 14-3-3eta or BiP, but may involve novel proteins identified by 2D-gel analysis. **A)** To examine if choline-mediated upregulation corresponds with changes in chaperone protein expression, western blot analysis was used to identify two candidate α4β2 chaperone proteins, 14-3-3, or the immunological heavy chain-binding protein, BiP, after cells were treated with either choline (Ch), hemichoinium-3 (HC30) or both. As seen in the results from these representative western blots, the changes in β2 expression, but stable α4 expression was as expected from previous experiments. In no case was any treatment-related change in BiP or 14-3-3eta measured relative to controls. **B)** To determine if other proteins are altered by choline and if identified changes are HC3-senstive, 2D-gel analysis of whole cell extract from stably transfected cells as identified were prepared and analyzed (see Text). 2D gels from control, choline or choline+HC3 treated cells are shown. Boxed regions (numbered 1–3) correspond to the same numbered enlargements shown in the next panel. **C)** Several spots that demonstrated changes in intensity or migration on 2D gels are shown at greater magnification (corresponding to the white boxes in B). The control (black arrow head) versus choline treatment (white arrow head) spots were removed from the blots, subjected to limited proteolysis and then subjected to sequencing using Mass-spectroscopy (see text). The identity of these three choline-responsive proteins shown is based upon protein-peptide sequence identity of 100% (sequences of proteolytic fragments are underlined and capital letters) and their sequence identity to the reference proteins noted taken from the NIH protein database (sequence match in bold). GTP-binding nuclear protein Ran (GI:5453555) maaqgepqvqfklvlvgdggtgkttfvkRHLTGEFEKkYVATLGVEVHPLVFHTNRgpikFNVWDTAGQEKfgglrDGYYIQAQCAIIMFDVTSRvtykNVPNWHRdlvrVCENIPIVLCGNKVDIKdrkvkaksivfhrkkNLQYYDISAKSNYNFEKPFLWLARkligdpnlefvampalappevvmdpalaaqyehdlevaqttalpdedddl//. Annexin A2, chain A (GI: 56966699) mstvheilcklslegdhstppsaygsvk AYTNFDAERDALNIETAIKtkGVDEVTIVNILTNRsneqrqdiafayqrrtkkelasalksalsghletvilgllktpaqydaselkasmkGLGTDEDSLIEIICSRtnqelqeinrvykemyktdlekdiisdtsgdfrklmvalakgrRAEDGSVIDYELIDQDAR.DLYDAGVKRkgtdvpkwisimtersvphlqkvfdryksyspydmlesirkevkgdlenaflnlvqciqnkplyfadrlydsmkgkgtrdkvlirimvsrsevdmlkirsefkrkygkSLYYYIQQDTKgdyqkallylcggdd//. Prefoldin 5 (GI:30583229) maqsinitelnlpqlemlkNQLDQEVEFLSTSIAQLK vvqtkyveakdclnvlnksnegkellvpltsssmyvpgkLHDVEHVLIDVGTGYYVEKtaedakdffkrkidfltkqmekiqpalqekhamkqavmemmsqtIQQLTALGAAQATAKA//. **D)** Subcellular distribution and expression of the cellular antigens identified by 2D-gel analysis and Mass spectroscopy using immunofluorescence of the 293 cells with protein-specific antibodies. All cells were treated, fixed, blocked and permeabilized and incubated with the indicated antibody overnight at 4°C. Cells were again washed and antibody binding revealed using an appropriate secondary antibody coupled to fluorescence. Random cell fields were then visualized and photographed as shown for each protein. Arrows points to representative cells of each field identify the typical appearance of antigen distribution under each condition.

A significant issue in this system to identify the cell signaling networks that lead to changes in β2 protein production and enhanced [^3^H]Eb density. One possibility is that a change in chaperoning of the receptor during assembly and transport through synthetic compartments leads to increased receptor expression [1,3]. Two candidate proteins that participate in this process are the immunological heavy chain-binding protein BiP [42] and 14-3-3eta [43]. To test the possibility that changes the expression of the 14-3-3eta or BiP correspond to upregulation related to PI3Kβ or Jak2 signaling, the expression levels of these chaperone proteins were measured. As above, subsequent to treatment of transfected 293 cells with choline, HC3 or choline and HC3 the whole cell lysates were analyzed using western blot. The typical results from 4 different experiments are shown in Fig 7A. Although the change in expression of β2 was as expected, in no case did treatment of the cells alter the expression of BiP or 14-3-3eta. The 14-3-3eta is shown because of its association to α4β2 [43], however no change in expression of other 14-3-3 proteins, as determined using additional antibodies or low isoform specificity, was observed (not shown). Thus, the expression of these candidate chaperone proteins did not vary with the treatments used it this study despite the change in α4β2 expression and upregulation.

To begin the search that will identify novel protein candidates that are responsive to regulation by choline and correspond with upregulation, we have initiated a proteomic approach using 2-D gel analysis. For this analysis three culture sets were prepared in parallel and the transfected 293 cells were treated with saline (control), choline or choline and HC3. At 24 hours post treatment, cells were harvested according to the instructions supplied by Applied Biomics Inc (see Methods) and then subjected to sample labeling and simultaneous 2-D gel analysis. Examples of the resulting 2D-gels, shown in Fig 7B, were then compared by both computer aided analysis (Applied Biomics) and visual inspection to identify proteins that exhibited a distinct quantitative or migration change in response to choline when compared to the control and in the presence of HC3. Examples of several proteins whose expression or migration on the gel was altered by choline and modified by HC3 were identified and their identity confirmed using proteolytic digestion followed by Mass spectroscopy and micro-sequencing. Protein identity was assigned through comparing sequences of several fragments to proteins of known sequence in the NCBI protein database. As shown in Fig 7C the migration of three proteins that were particularly sensitive to choline and exhibited HC3-sensitivity are expanded to show the selected spots. The identity of the proteins, Ran-GTPase (GI: 48734884), annexinA2 (GI: 56966699), and prefoldin5 (GI:22202633), was based upon 100% sequence identity for multiple peptides of each spot. The Ran-GTPase exhibited a decrease in apparent molecular weight following choline treatment and this was largely inhibited by HC3. Similarly, annexin2 also changed migration in the SDS-PAGE phase towards an apparent reduced molecular weight. This choline-mediated shift was also inhibited by HC3. In contrast, prefoldin 5 expression was strongly induced by an HC3-sensitive choline-mediated mechanism.

Antibodies to each of these proteins were obtained to determine if the proteins identified by 2D-gel also exhibited a change in cellular location or intensity in response to treatment. Cells were again prepared only for this assay (see Methods for details) they were grown on glass coverslips coated with poly-lysine (Methods). At 24 hours post treatment cells were fixed, blocked and permeabilized and incubated with the indicated antibody overnight at 4°C. Cells were again washed and antibody binding revealed using an appropriate secondary antibody (Methods). Random cell fields were then photographed and samplings of these are show in Fig 7D for each protein. Each of these proteins exhibits a change in their respective subcellular staining patterns following choline or choline and HC3 treatment. Notable was the robust change in protein distribution of Ran revealed by the loss of nuclear staining after choline treatment. The presence of HC3 produces an intermediary expression pattern although there is a reduction of signal in this cell group compared to the choline treatment alone (Fig 7D). This compares well to the 2D gel where the expression of the spot identified as Ran is reduced in the choline treated group. Although the function of Ran is most often viewed in nucleocytoplasmic transport [44], this GTPase can also form complexes with proteins in the cytoplasm to regulate phagocytosis [44], similar to one function of PI3Kβ[45], and it interacts with binding partners to Jak2 [46]. Also, PI3Kβ has been implicated in directly controlling nuclear membrane integrity and transport through upstream effects on Ran activation [47]. For annexin2, choline-treated cells exhibit a stronger staining pattern and more peri-nuclear localization than does the control whose expression appears to be more in vesicular-like structures throughout the cytoplasm. This apparent intensification of signal and possible redistribution was inhibited by HC3. The finding that annexin2 is modified in this system corresponds to reports of its role in multiple cellular processes including endocytosis, exocytosis, ion channel conductance and membrane organization, and regulation of LDL receptors [48]. Further, annexin2 can directly bind to choline [49]. Notably, upon stimulation of chromaffin cells with nicotine, annexin2 undergoes phosphorylation, consistent with the shift in spot migration observed in the 2D gel analysis, and it exhibits a subcellular redistribution from the cytosol to a Triton X-100 insoluble fraction [50,51]. Finally, prefoldin-5 exhibits a strong increase in expression in choline treated cells that is inhibited by HC3. This is consistent with 2D gel analysis. Prefoldins are a family of ubiquitously expressed co-chaperone proteins that contribute to folding of nascent proteins and their subsequent transport [52]. In particular, the prefoldin 5 is related to cytoskeleton integrity [53] and its loss in mice corresponds with multiple neurodegenerative and central abnormalities [54].

Collectively, the identification of these proteins and possibly others not yet further examined suggest novel avenues to examine how choline modifies the cellular processes leading to upregulation of the [^3^H]Eb sites reflecting increased α4β2 expression. Further, since these proteins have not been related directly to α4β2 receptor expression regulation, future studies to determine if they participate directly in this process and which cell-signaling cascade dominates in their regulation of expression could prove to offer novel off-receptor cellular targets to regulate this process associated with addiction.

## Discussion

In previous reports we identified multiple non-ligand mechanisms associated with the regulation of high affinity [^3^H]Eb density binding-sites that reflect changes in α4β2 receptor subunit expression by HEK cells stably transfected with these subunits [9,10,14]. In this study the efficacy of a wide range of small molecule inhibitors targeting key intracellular signaling pathways were evaluated for their participation in the cellular processes underpinning this process of upregulation. Several key insights extend our previous studies to reveal novel interactions between the PI3K, Jak2 and p38Mapk networks in the control of [^3^H]Eb density corresponding with an increase in β2 subunit protein. This includes the demonstration that the class 1 PI3Kβ activity is a constitutive negative modulator of p38Mapk and α4β2 receptor upregulation since inhibition by PI828 is sufficient to produce modest upregulation independent of receptor ligand and Akt phosphorylation. Also revealed in this study was that TNFα-mediated enhancement of upregulation and HC3-sensitive choline-upregulation both include Jak2 activation and subsequent positive modulation of p38Mapk.

In both the CNS and peripheral cells choline is a precursor to acetylcholine production, but choline phosphorylation by CK is also a key precursor to production of phosphocholine, a major membrane constituent. One implication of this could be of relevance to the elegant studies by the Green group [3,7,8] who have used conditions of mild membrane disruption to demonstrate that the upregulation process also harbors a fast component where changes in receptor conformation lead to increased ligand-affinity. Unfortunately in this study the methods used to solubilize receptors fail to retain this delicate conformational change. Thus our measurements are restricted to measuring the slower component of upregulation that in this model system is related to increased β2 production [9,10,14]. Nevertheless, the manipulation of the membrane phospholipids through choline-dependent mechanisms, possibly through feedback-mechanism associated with metabolic pool accumulation of choline products [55], would be a valuable tool for measuring finer points regarding how the membrane structure influences the ligand-binding as well as the state of receptor expression.

These results do suggest novel ways through which α4β2 upregulation can be manipulated by ligand-independent intracellular signaling pathways associated with PI3K, p38Mapk and Jak2 pathways. These signaling pathways are most often associated with immune functions and environmental cues as associated with diet. An interaction already reported between Jak2 and α4β2 is noteworthy since the inhibition of Jak2 using AG-490 leads to a decreased efficacy of the anti-inflammatory impact by α4β2 and this could be through a direct signaling interaction between α4β2 and Jak2 [29]. Nevertheless, there remains the possibility of overlap in these pathways that in part could reflect the need to use a relatively high concentration of AG-490 (100 μM) to inhibit the HC3-sensitive CK pathway. Although all Jak2 inhibitors impacted upon upregulation, cross-interaction with other kinases that will contribute to this result cannot be ruled-out. In this context, the identification of functional mutations in Jak2 that are more common in smokers than nonsmokers [56] is intriguing. Further, modifications to receptor expression by choline, a dietary component and acetylcholine metabolic product produce additional changes to receptor expression [9,10,14]. In terms of the α4β2 receptor, these types of interactions appear to be tightly controlled and are likely to be highly cell specific both through the nature of the cell signaling network and the inclusion of additional nAChR subunits into the α4β2 complex. This includes alpha5, which alleviates upregulation to ligand and TNFα [14,15] and possibly other subunits such as α6 or β3 [3,6,7,57] that in combination with α4 and β2 exhibit substantially altered upregulation properties relative to α4β2 alone. What is evident is that in addition to the complexity of upregulation cell biology, the control of α4β2 receptors density is likely to be highly cell specific and far more integrated into the cellular signaling networks that are responsive to exogenous, inflammatory and metabolic agents than has been previously believed.

**Fig 8:**
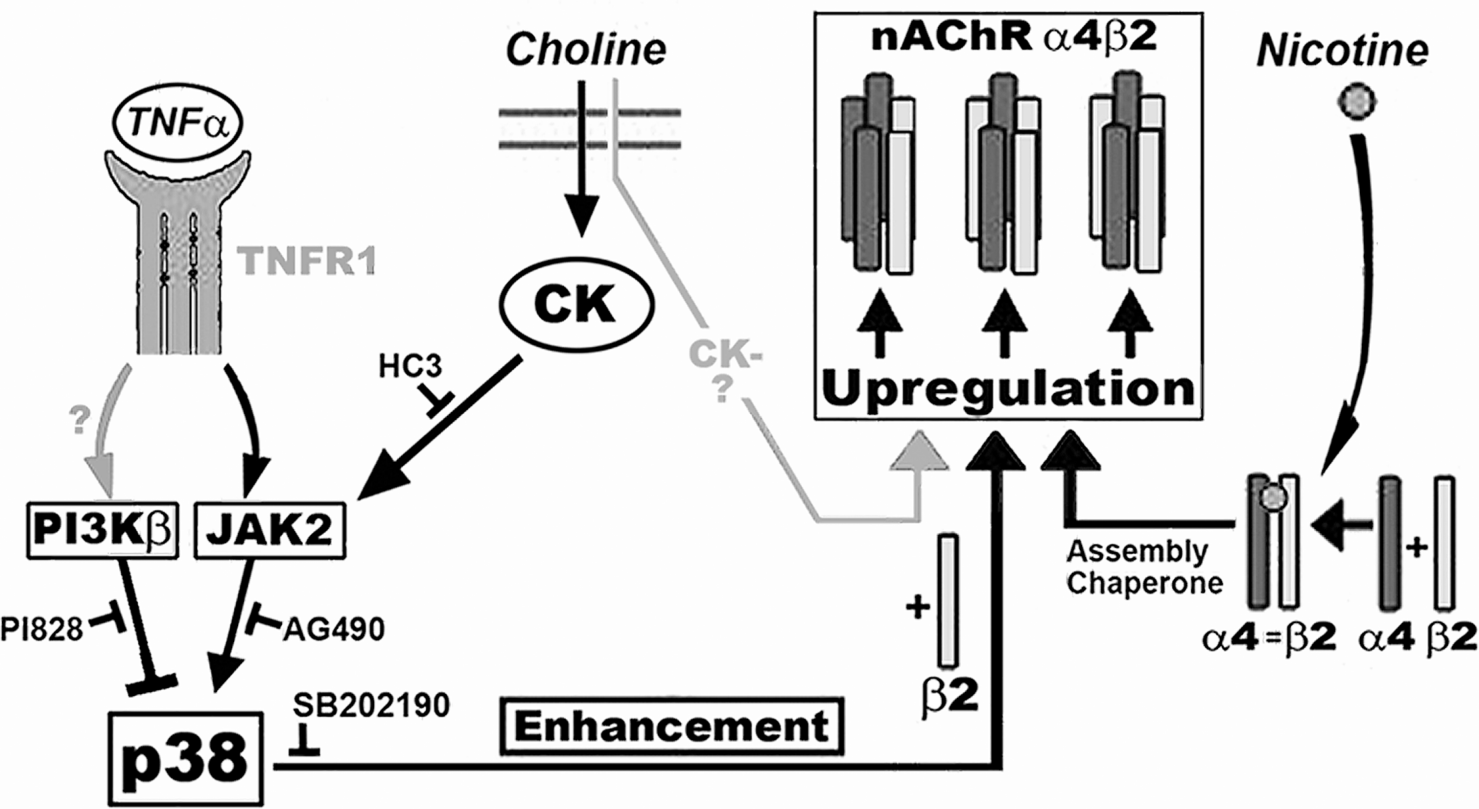
Diagram summarizing the findings of this study. Assembly of mature α4β2 nAChRs from a precursor pool of individual subunits can be upregulated by nicotine, possibly acting as a chaperone to increase α4+β2 subunit association and subcellular transport. The mechanisms through which choline and TNFα influence this process is to ultimately increase β2 subunit production (favoring pentameric α4+β2 assembly) through a p38MAPK mechanism that is inhibited by SB202190. The activity of p38MAPK is balanced by constitutive inhibition by PI3kβ (inhibited by PI828) and promoted by an increase in Jak2 kinase activity (inhibited by AG490). Jak2 activation is stimulated by choline acting through a choline kinase (CK) pathway that is distinguished by hemicholinium3-sensitivity (HC3) pathway or signaling by the pro-inflammatory cytokine tumor necrosis factor alpha (TNFα) acting through its receptor, TNFR1. The choline mediated upregulation mechanism through the HC3-insensitve pathway (CK-, in light grey) is not yet characterized but also produces upregulation through increased β2 production.

When our results are placed into the context of cellular regulation there is the suggestion that quantitative modulation is a normal aspect of regulating α4β2 receptor numbers through the convergence of signaling networks that are responsive to diet (e.g., choline) and inflammation (Fig 8). This also suggest that upregulation to nicotine reflects an example of the impact by an exogenous agent that imbalances this finely-tuned regulatory interaction. Thus, continuous signaling through PI3Kβ is an important brake on producing high-affinity α4β2 sites whereas positive modulation through p38Mapk activation as during a pro-inflammatory event would be controlled by the TNFα/TNFR1 signaling intensity to Jak2 and enhanced activation of p38Mapk. Thus in terms α4β2 upregulation, p38Mapk serves as a metabolic ‘coincidence’ detector where convergence of Jak2 and PI3Kβ signaling is weighted and an outcome determined. The possibility that TNFR1 recruits and interacts directly with Jak, PI3K and p38Mapk has been proposed [58], and a direct interaction between PI3K and p38Mapk signaling is known [59]. The potential for TNFR1 forming the focus of such a cell-specific signaling complexes, especially recruitment of Jak2 into a complex that is sensitive to TNFα, is attractive [58,60–63]. Collectively, the enhancement of upregulation would include a summation of contributions by precursor pool status of α4 and β2, TNFR1/Jak2 upstream signaling to p38Mapk, signaling through the choline HC3-sensitive pathway to Jak2 and the direct disinhibition of p38Mapk by inhibiting constitutive PI3Kβ activity (Fig 8). While cross-talk between different cellular signaling pathways is common, they are also highly cell specific. Thus, confirmation of the relevance of this scheme to neuronal upregulation will be important towards understanding how this fits into the physiology contributing to the addiction phenotype. What does seem likely is that this important nicotinic receptor is subjected to significant modulation during periods of inflammatory illness and metabolic stress. Novel approaches to interfere with nicotine–mediated α4β2 upregulation are likely to be derived from a better understanding of these interactions.
